# Lessons Learned from *In Vivo* Studies of a Viral Noncoding RNA

**DOI:** 10.1128/mSphere.00026-16

**Published:** 2016-03-02

**Authors:** Rodney P. Kincaid, Christopher S. Sullivan

**Affiliations:** Department of Molecular Biosciences, Center for Systems and Synthetic Biology, LaMontagne Center for Infectious Disease, Institute for Cellular and Molecular Biology, University of Texas at Austin, Austin, Texas, USA

**Keywords:** Epstein-Barr virus, Kaposi's sarcoma-associated herpesvirus, gammaherpesvirus, latency, microRNA, noncoding RNA, viral dissemination

## Abstract

Despite increasing interest in the biology of noncoding RNAs (ncRNAs), few functions have been uncovered for viral ncRNAs *in vivo*. In their recent article in *mSphere*, Feldman and colleagues [E. R. Feldman et al., *mSphere* 1(2):e00105-15, 2016, doi:10.1128/mSphere.00105-15] demonstrate a highly specific activity of a gammaherpesviral ncRNA in viral dissemination and establishment of latent infection. Their work highlights several interesting features that should be informative to future studies of viral ncRNA.

## TEXT

The field of non-protein-coding regulatory RNA, or noncoding RNA (ncRNA), has gained much momentum in the last decade. ncRNA has been implicated in important aspects of all domains of life. In eukaryotes, ncRNAs derive from a collection of numerous different classes of RNAs with lengths ranging from 20 nucleotides to kilobases. Many ncRNAs have been implicated in transcriptional and posttranscriptional control of gene expression, with the microRNA (miRNA) class of ncRNAs being among the best characterized. Although often overlooked, some of the first well-characterized ncRNAs are of viral origin (1, 2). These include the adenoviral virus-associated RNAs (VA RNAs) and the herpesviral HSUR and EBER RNAs, each being first described over 25 years ago (3 – 5). Despite being among the best characterized, important questions remain even about these viral ncRNAs, including a full detailed mechanistic understanding of their molecular activities as well as their relevant functions *in vivo*. In fact, the functions of the majority of viral ncRNAs are completely unknown, and very few have been studied in the context of infection *in vivo*.

Gammaherpesviruses replicate and persist predominantly in lymphoid cells and are etiologic agents of several types of cancers (6). Typically, these cancers are associated with an immunocompromised host. In humans, the two known gammaherpesviruses are Epstein-Barr virus (EBV) and Kaposi’s sarcoma-associated herpesvirus (KSHV), and both are associated with lymphoid and solid tumors (7). Most if not all herpesviruses encode ncRNAs. EBV and KSHV both encode miRNAs, small RNAs that dock to mRNAs and direct decreased steady-state mRNA levels and inhibition of translation. In addition, EBV encodes EBERs, RNA polymerase III (Pol III)-transcribed ncRNAs that are the most abundant viral transcripts in infected cells (up to 10^7^ copies/cell) (4). Because infection with both EBV and KSHV is species specific, there remains a limited understanding of their life cycles *in vivo*. In contrast, studies of the murine gammaherpesvirus 68 (MHV68) can readily take advantage of well-established laboratory *in vivo* mouse experimental systems. Like KSHV and EBV, MHV68 infects B cells, is associated with pathogenesis in immunocompromised hosts, and encodes abundant ncRNAs during lytic and latent infection.

MHV68 encodes a unique class of ncRNAs that are now known as tRNA-microRNA-encoded RNAs (TMERs). The TMERs were first reported as a cluster of eight nonaminoacylated viral transfer RNAs (vtRNAs) that were expressed in both latently and lytically infected cells (8). However, in 2005, Pfeffer et al. identified miRNAs encoded immediately following the MHV68 vtRNAs (9). It was later confirmed that the vtRNAs and viral miRNAs are cotranscribed by RNA Pol III (10, 11). The primary TMER transcripts are processed by the host enzyme tRNase Z, which results in the separation of the vtRNAs from the pre-miRNA structures (11). This unusual arrangement allows the TMER genes to produce two different classes of ncRNAs. Previous studies from some of the same labs involved in this current work of Feldman et al. (12) mostly focused on TMER mutant viruses defective for expression of numerous viral ncRNAs (13, 14). These studies showed that TMERs are dispensable for infection in cultured cells but can have activities *in vivo* related to both pathogenesis and latency. These results suggest that the MHV68 TMERs are multifunctional ncRNAs and that further studies of individual TMERs are required to decode their full repertoire of functions.

**FIG 1:**
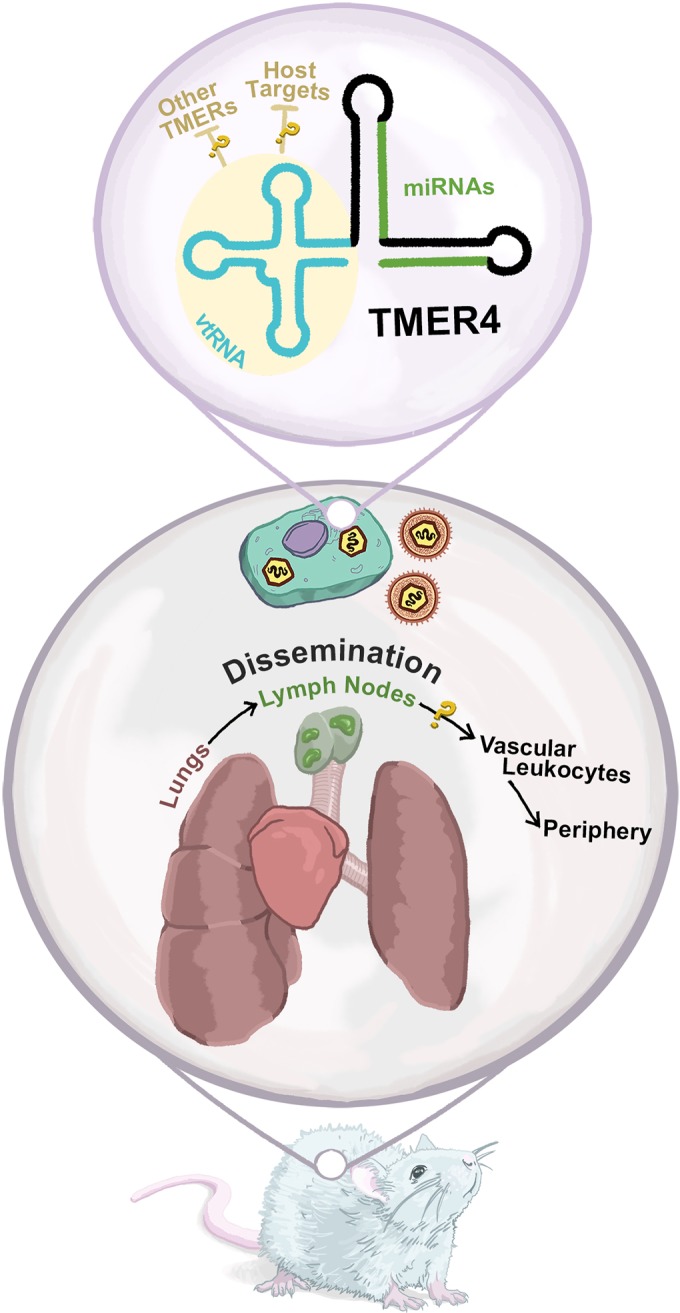
TMER4, a murine gammaherpesviral ncRNA, likely functions to promote dissemination from lung-draining lymph nodes to vasculature leukocytes and subsequent peripheral sites of infection. The study of Feldman et al. (12) identified a new activity for a viral ncRNA. Yellow question marks illustrate some interesting molecular and physiological questions raised by their study. The tRNA-like (vtRNA) and pre-miRNA portions of the TMER ncRNA are indicated.

In their most recent report, Feldman and colleagues uncover an interesting phenotype for a deletion mutant virus that abrogates a single TMER, TMER4 (12). TMER4 is shown to be important for establishing latent infection. While a worthy observation in itself, it is particularly notable that the authors demonstrated that this defect can be bypassed by utilizing a different, less natural route of inoculation. While mice intranasally (i.n.) inoculated with the TMER4 mutant virus had a substantially reduced number of latently infected B cells, mice that were inoculated intraperitoneally (i.p.) directly into the abdominal cavity showed no defect. The authors then demonstrated that TMER4 is not required for acute lytic infection. Furthermore, there were no defects in infectious virus levels or the fraction of latently infected cells in the lung-draining lymph nodes, the tissue that is the most likely portal for viral dissemination from the lung to circulating leukocytes. In contrast, at a time postinfection when wild-type virus is detectable in white blood cells but not yet in peripheral sites, such as the spleen, the TMER4 mutant virus showed a strong defect in viral genome copy number in leukocytes. Based on results of a painstaking series of experiments and a process of elimination, the authors compellingly argue that TMER4 functions to promote virus dissemination. Thus, from the lung-draining lymph nodes, viruses lacking TMER4 cannot effectively gain access to the vasculature leukocytes and subsequent peripheral sites of infection (Fig. 1).

In addition to the observation that the route of inoculation can profoundly affect phenotype, this work uncovered several other findings that are likely instructive for other viral ncRNAs. First, TMERs are “polyfunctional.” Like adenovirus VA RNAs that have functions ascribed to the longer ncRNAs as well as their miRNA derivatives (15), the longer TMERs have functions that are separate from their miRNA derivatives. Here, Feldman and colleagues demonstrate that the miRNA derivatives of the TMER4 are dispensable for its activities in dissemination and establishment of latency. Second, adding an interesting twist, it has been previously shown that mutation of all MHV68 miRNAs results in only a mild defect in establishing latency *in vivo* (13). This argues that one or more TMERs may have deleterious activities that are countered by TMER4.

Feldman and colleagues have presented a methodical study that uncovers one of the few *in vivo* activities known for a viral ncRNA. However, many unanswered questions remain. Is it possible that lung-draining lymph node-independent routes exist to allow peripheral infection? Does TMER4 function only to regulate the deleterious effects of another TMER(s)? What is the relevant function of TMER4 miRNAs? And, perhaps most interestingly, what is the molecular activity of TMER4 that accounts for its role in dissemination? The lessons learned from this study are humbling and should be of value to the design of future studies of viral ncRNA. Not only would the phenotype of the TMER4 mutant virus have been missed in cultured cells, but it would have been missed *in vivo* if an alternative route of inoculation or a less surgical mutant virus design strategy had been used. Such work illustrates the power of fully infectious murine viral experimental systems. It remains to be seen if human-pathogenic viruses utilize ncRNAs to promote similar dissemination-dependent persistent infections.
